# LINGUISTIC PROFILES OF PARAGRAPH RECALL PREDICT DEPRESSION IN OLDER ADULTS

**DOI:** 10.1093/geroni/igad104.0047

**Published:** 2023-12-21

**Authors:** Seho Park, Nicole Roth, Paola Sebastiani, Stephanie Cosentino, Stacy Andersen

**Affiliations:** Boston University Chobanian & Avedisian School of Medicine, Boston, Massachusetts, United States; Boston University School of Medicine, Boston, Massachusetts, United States; Tufts Medical Center, Boston, Massachusetts, United States; Columbia University Medical Center, New York City, New York, United States; Boston University Chobanian & Avedisian School of Medicine, Boston, Massachusetts, United States

## Abstract

Our previous work identified linguistic markers from paragraph recall that predicted cognitive impairment. However, many features were related to psychological constructs, and mood disorders, such as depression, are also associated with cognitive impairment. Therefore, we aimed to identify linguistic markers that are associated with depression and examine their overlap with those for cognitive impairment. We used Linguistic Inquiry Word Count, a natural language processing tool, on Logical Memory transcriptions from the Long Life Family Study. Depression status was positive for participants with a self-reported depression diagnosis or high depressive symptoms (i.e., a CESD-10 score≥10) at testing. We used logistic regression models with Generalized Estimating Equations adjusted by age, sex, education, and within-family relatedness to identify linguistic features associated with depression status (Bonferroni-corrected threshold of p<.001). The sample included 61 participants with evidence of depression and 539 participants without (mean age=72.1±10.9 years, female=63%). A lower percentage of verb use in delayed recall was significantly associated with depression (Odds Ratio [OR]=0.71, CI 0.55-0.93, p=.0001). Additionally, 3 immediate recall features (verbs, OR=0.71; prepositions, OR=1.30; differentiation words, OR=1.30) and 5 delayed recall features (function words, OR=0.68; prepositions, OR=1.50; future tense, OR=0.48; cognitive process words, OR=0.78; past tense, OR=0.74) were significant at a nominal level (p=.009 to p=.035). We identified linguistic features from paragraph recall responses that predict depression and were largely distinct from those associated with cognitive impairment as only two features overlapped. Linguistic analyses of spoken responses may help us understand contributors to test performance and distinguish between cognitive impairment and depression.

